# Preliminary Evidence of Orthostatic Intolerance and Altered Cerebral Vascular Control Following Sport-Related Concussion

**DOI:** 10.3389/fneur.2021.620757

**Published:** 2021-04-09

**Authors:** Morgan L. Worley, Morgan C. O'Leary, James R. Sackett, Zachary J. Schlader, Barry Willer, John J. Leddy, Blair D. Johnson

**Affiliations:** ^1^Center for Research and Education in Special Environments, Department of Exercise and Nutrition Sciences, School of Public Health and Health Professions, University at Buffalo, Buffalo, NY, United States; ^2^Human Integrative Physiology Laboratory, Department of Kinesiology, School of Public Health, Indiana University, Bloomington, IN, United States; ^3^Department of Psychiatry, Jacobs School of Medicine and Biomedical Sciences, University at Buffalo, Buffalo, NY, United States; ^4^UBMD Department of Orthopaedics and Sports Medicine, University at Buffalo, Buffalo, NY, United States

**Keywords:** mild traumatic brain injury, cerebral blood flow, blood pressure, baroreflex, autonomic function

## Abstract

Concussions have been shown to result in autonomic dysfunction and altered cerebral vascular function. We tested the hypothesis that concussed athletes (CA) would have altered cerebral vascular function during acute decreases and increases in blood pressure compared to healthy controls (HC). Ten CA (age: 20 ± 2 y, 7 females) and 10 HC (age: 21 ± 2 y, 6 females) completed 5 min of lower body negative pressure (LBNP; −40 mmHg) and 5 min of lower body positive pressure (LBPP; 20 mmHg). Protocols were randomized and separated by 10 min. Mean arterial pressure (MAP) and middle cerebral artery blood velocity (MCAv) were continuously recorded. Cerebral vascular resistance (CVR) was calculated as MAP/MCAv. Values are reported as change from baseline to the last minute achieved (LBNP) or 5 min (LBPP). There were no differences in baseline values between groups. During LBNP, there were no differences in the change for MAP (CA: −23 ± 18 vs. HC: −21 ± 17 cm/s; *P* = 0.80) or MCAv (CA: −13 ± 8 vs. HC: −18 ± 9 cm/s; *P* = 0.19). The change in CVR was different between groups (CA: −0.08 ± 0.26 vs. HC: 0.18 ± 0.24 mmHg/cm/s; *P* = 0.04). Total LBNP time was lower for CA (204 ± 92 s) vs. HC (297 ± 64 s; *P* = 0.04). During LBPP, the change in MAP was not different between groups (CA: 13 ± 6 vs. HC: 10 ± 7 mmHg; *P* = 0.32). The change in MCAv (CA: 7 ± 6 vs. HC: −4 ± 13 cm/s; *P* = 0.04) and CVR (CA: −0.06 ± 0.27 vs. HC: 0.38 ± 0.41 mmHg/cm/s; *P* = 0.03) were different between groups. CA exhibited impaired tolerance to LBNP and had a different cerebral vascular response to LBPP compared to HC.

## Introduction

Nearly 3.8 million individuals sustain a sport-related concussion every year in the United States and many more are sustained *via* car crashes, falls, and military action (1). It has been proposed that following head trauma there is a neurometabolic cascade that ensues which may influence autonomic nervous system function as well as cerebral blood flow and cerebral vascular function (2, 3). Accumulating evidence has indicated that individuals who have sustained a concussion exhibit autonomic dysfunction (4) and altered cerebral blood flow (5–7). For instance, asymptomatic post-mild traumatic brain injury (mTBI) patients exhibited reduced cardiac baroreflex sensitivity (cBRS) at rest (4, 8) and upon standing compared to healthy controls (4). Similarly, cerebral autoregulatory dysfunction was found in acutely concussed athletes (i.e., ice hockey and American football) during squat-to-stand maneuvers (9) and in active professional boxers with mild to moderate chronic brain injury using the thigh cuff deflation technique (10). These investigations have studied patients at rest (4, 8, 11) or using experimental techniques that induce only acute decreases in blood pressure to assess cBRS (4) and cerebral vascular function (9, 10). However, other common stressors that occur outside of the lab, such as mental stress (12–14) and exercise (15), can cause acute increases in blood pressure. These increases in blood pressure might contribute to an exacerbation of concussion symptoms secondary to impaired blood pressure control and/or abnormal cerebral vascular function. Thus, it is important to assess cerebral vascular function and the control of blood pressure during both decreases and increases in blood pressure.

Lower body negative pressure (LBNP) and lower body positive pressure (LBPP) are physiological stressors that can be used to acutely decrease and increase arterial blood pressure, respectively. These techniques can therefore be used to investigate both cerebral blood flow responses and autonomic baroreflex function in individuals who have sustained a concussion. Unlike the sit-to-stand tests, which have been used to demonstrate impaired dynamic cerebral vascular function in concussed patients (9, 16), LBNP and LBPP do not activate the vestibular system. Vestibular system disturbances are common following a concussion and can lead to symptom exacerbation, primarily dizziness, which can confound cBRS and cerebral blood flow outcomes during assesments that involve postural changes (17). To the best of our knowledge, no one has experimentally induced a rise in blood pressure above resting values to examine cBRS and cerebral vascular function in symptomatic concussed athletes. Thus, LBNP and LBPP protocols are excellent techniques to assess alterations in blood pressure in concussion patients because these techniques circumvent the possible influence of vestibular activation and allow for a more clear interpretation of the data. Furthermore, sit-to-stand tests examine cerebral blood velocity responses to rapid oscillations in blood pressure (9) whereas LBPP acutely increases and sustains a rise in blood pressure. The combined use of LBNP and LBPP can better illuminate the baroreflex and cerebral vascular responses to changes in blood pressure in patients who have sustained a concussion. To this end, the primary aims of our study were to investigate cerebral vascular function and cardiac-baroreflex sensitivity during acute changes in arterial blood pressure in symptomatic concussed college athletes. We hypothesized that compared to healthy controls, concussed athletes would (1) have a greater reduction in cerebral blood velocity during LBNP, (2) have lower cBRS during LBNP, (3) have a greater increase in cerebral blood velocity during LBPP, and (4) have lower cBRS during LBPP.

## Methods

### Participants

Ten concussed college athletes (CA) (7 females; age: 20 ± 2 y; height: 177 ± 10 cm; weight: 77 ± 10 kg; BMI: 25 ± 3 kg/m^2^) and 10 healthy controls (HC) (6 females; age: 21 ± 2 y; height: 174 ± 7 cm; weight: 76 ± 15 kg; BMI: 25 ± 3 kg/m^2^) completed the study. Using feasibility data, we calculated the *a priori* sample size based on the cardiovascular responses to a sympathetic stressor in 10 CA and 10 HC. We calculated we would need 8 participants in each group using an effect size (*f* ) of 0.38, alpha of 0.05, and power of 0.80 to observe a significant group x time interaction effect. CA completed the study within 10 days of concussion (5 ± 3 days) diagnosis by a University at Buffalo physician and while symptomatic (SCAT-5 score: 23 ± 36; range: 2–85). All concussions were sport-related and diagnosed using the most recent Concussion in Sport Group guidelines (18), including history, concussion symptoms linked to a head injury *via* the Post-Concussion Symptom Inventory (19), or injury to another part of the body with forced transmission to the head, and impairments on a concussion-focused physical exam (20). The CA group self-reported to not have had a concussion within the previous year (with the exception of the most recent concussion). All CA participated in collegiate sports. Female athletes participated in rowing (*n* = 1), volleyball (*n* = 2), soccer (*n* = 2), and basketball (*n* = 2). Male athletes participated in football (*n* = 1), wrestling (*n* = 1), and lacrosse (*n* =1). The HC group self-reported to be physically active on most if not all days of the week *via* the International Physical Activity Questionnaire and self-reported no concussions within the previous year. We used healthy controls that did not participate in collegiate athletics to avoid the unknown effects of repetitive subconcussive head impacts on physiological function. Participants were excluded if they had a Glasgow Coma Score of <12, focal neurological deficit, used medications that affect blood pressure or the autonomic nervous system (beta-blockers, alpha-blockers, angiotensin converting enzyme inhibitors, angiotensin receptor blockers); existing autonomic, cardiovascular, metabolic, respiratory, or endocrine disorder; major depression; pregnancy; or breastfeeding. The concussed athletes were studied while still reporting post-concussion symptoms based on a symptom severity inventory (Sport Concussion Assessment Tool−5th Edition (SCAT-5)) (18). This 22-question Likert scale questionnaire assesses a variety of symptoms (None = 0, Moderate = 3, Severe = 6). The sum of these responses was calculated to express total symptom severity (0–132). All participants were fully informed of the experimental procedures prior to providing informed, written consent. The study was approved by the Institutional Review Board at the University at Buffalo and was performed in accordance with the standards set by the latest revision of the Declaration of Helsinki.

### Experimental Approach

Participants completed one study visit that consisted of an LBNP protocol and an LBPP protocol. Participants reported to the laboratory after abstaining from alcohol, exercise, and caffeine for 12 h, and food for 2 h. Women of childbearing potential voided their bladder for a urine pregnancy test and reported the start date of their last menstrual cycle. It is important to note that menstrual cycle phase might influence cerebral vascular function (21, 22) and baroreflex function (23) however, we were unable to control for the menstrual cycle due to testing participants within the first 10 days of concussion diagnosis and menstrual cycle phase was not a focus of our investigation. After voiding their bladder, participants assumed the supine position and their lower body was sealed into an airtight chamber at the level of the iliac crest using a neoprene skirt prior to being instrumented to continuously record beat-to-beat blood pressure (photoplethysmography), heart rate (3-lead electrocardiogram), and end-tidal CO_2_ tension (capnography). Right MCAv was measured *via* transcranial Doppler sonography using a 2 MHz probe (DWL USA, Inc., Germany, Europe) while using insonation techniques described in detail by Willie et al. (24).

After instrumentation, participants completed a 5-min baseline period before commencement of the protocols. Prior to and following the LBNP and LBPP protocols, concussed athletes reported their concussion symptom severity using a visual analog scale (VAS; 0 = feel terrific, no symptoms; 10 = feel terrible, worst I ever felt). The order of the protocols was randomized by a research team member prior to enrollment using Excel. Protocols were separated by 5 min of recovery and 5 min of quiet resting baseline (>10 min between protocols). Eight CA (5 women) and six HC (2 women) completed LBPP first. During the LBNP protocol, an acute decrease in blood pressure was achieved by applying 40 mmHg of LBNP for 5 min (25–27). If systolic blood pressure sustained a fall below 80 mmHg along with a precipitous decrease in heart rate, or if participants reported any symptoms of pre-syncope (e.g., sweating, tunnel vision, nausea, dizziness), the LBNP protocol was terminated (28). During the LBPP protocol, an acute increase in blood pressure was achieved by applying 20 mmHg of LBPP for 5 minutes (29–31). The LBPP protocol was terminated if participants desired to self-terminate or if systolic blood pressure sustained an increase above 180 mmHg.

### Data Acquisition

Heart rate (1,000 Hz), beat-to-beat blood pressure (1,000 Hz), MCAv (1,000 Hz) and end-tidal CO_2_ tension (62.5 Hz) were continuously recorded using a data acquisition system (Biopac MP150, AcqKnowledge 4.2.0, Goleta, CA). Stroke volume was estimated using Modelflow (32) and cardiac output was calculated as the product of heart rate and stroke volume. Cerebral vascular resistance (CVR) was calculated as MAP divided by MCAv. Mean values were extracted for the last 2 min of baseline and for every minute of the LBNP and LBPP protocols. The absolute change from baseline values were calculated for each minute of LBNP and LBPP. Total LBNP time was recorded (s) as an indicator of orthostatic tolerance. Due to early termination of LBNP, only participants that completed at least 3 min of the 5 min protocol were included in the anaylsis for cBRS (see below) (33). Total LBPP time was not analyzed since all participants completed the protocol (300 s). One healthy control was excluded from MCAv and cerebral vascular resistance analysis during LBNP due to excessive noise (>10 s of excluded data) (34).

### Assessment of Cardiac-Baroreflex Sensitivity (cBRS)

Raw data files of ECG and beat-to-beat blood pressure were used for offline analysis of cardiac-baroreflex sensitivity (WinCPRS, Absolute Aliens Oy, Turku, Finland). Three minute and five minute recordings for LBNP and LBPP were included for analysis, respectively. Participants were excluded from LBNP analysis of cBRS if the test was terminated prior to reaching 3 min of negative pressure [excluded concussed athletes (*n* = 3); excluded healthy controls (*n* = 2)] (33). The ECG and blood pressure waveforms were visually inspected for artifact and ectopic beats. We used the sequence method to calculate cBRS (35, 36). Four successive increases or decreases in systolic blood pressure (SBP) (±1 mmHg) that corresponded with changes in R-R interval (RRI) (±5 ms) were identified. Sequences were deemed valid if the *R*^2^-value calculated from individual linear regression analyses between SBP and RRI was ≥0.85 (37). The time delay between SBP and RRI was set to 1 beat (38). Sequences were determined separately for Up-cBRS (i.e., concurrent increases in RRI and SBP) and Down-cBRS (i.e., concurrent decreases in RRI and SBP decreased).

### Statistical Analysis

We used unpaired *t*-tests to determine if resting baseline values for cardiovascular and cerebrovascular variables and total LBNP time were different between CA and HC. We used two-way repeated measures ANOVAs with time as a within-subject effect and group as a between-subject effect to compare responses for LBPP between CA and HC. Mixed-effects ANOVAs were used to compare responses between CA and HC during LBNP due to missing data from terminating the test early (*n* = 9 total events). All ANOVA assumptions were visually verified (Q-Q plots and residual plots) prior to model interpretation and all assumptions were met and no corrections were made. If an ANOVA revealed a significant interaction or main effect (39), we used Bonferroni *post-hoc* analyses to determine where differences occurred. Additionally, if the Bonferroni *post-hoc* analyses revealed a difference between groups, we calculated the effect size, Cohen's *d*. A Kaplan-Meier survival analysis was used to assess the survival rate between CA and HC during the LBNP protocol. Lastly, we used paired *t*-tests to compare concussion symptom severity in the CA group before and after each protocol. Statistical analyses were performed using Prism software (version 8, GraphPad Software, La Jolla, CA). Statistical significance was set at *P* ≤ 0.05. Exact *P*-values are reported where appropriate. Values are reported as mean ± SD of the change from baseline.

## Results

### Lower Body Negative Pressure

There were no differences between groups for all variables at baseline (Table 1).

**Table 1:** Baseline LBNP and LBPP-values for concussed athletes (CA) and healthy controls (HC).

	**LBNP**			**LBPP**		
	**CA**	**HC**	***P*-value**	**CA**	**HC**	***P*-value**
Mean arterial pressure (mmHg)	93 ± 10	95 ± 8	0.521	90 ± 7	94 ± 9	0.305
Systolic blood pressure (mmHg)	130 ± 16	133 ± 11	0.710	128 ± 17	129 ± 9	0.872
Diastolic blood pressure (mmHg)	74 ± 6	71 ± 9	0.473	73 ± 7	70 ± 6	0.275
Heart rate (bpm)	59 ± 10	63 ± 10	0.317	57 ± 7	65 ± 12	0.109
Stroke volume (mL)	92 ± 18	96 ± 31	0.774	92 ± 7	93 ± 29	0.943
Cardiac output (L/min)	5 ± 1	6 ± 2	0.171	5 ± 1	6 ± 2	0.297
End-tidal CO_2_ tension (mmHg)	45 ± 2	45 ± 3	0.978	44 ± 3	45± 3	0.173
MCAv (cm/s)	63± 16	64 ± 11	0.830	57 ± 19	63 ± 10	0.413
Cerebral vascular resistance (mmHg/cm/s)	1.5 ± 0.4	1.5 ± 0.3	0.840	1.7 ± 0.6	1.5 ± 0.3	0.457

#### Cardiovascular Responses

**Figure 1:**
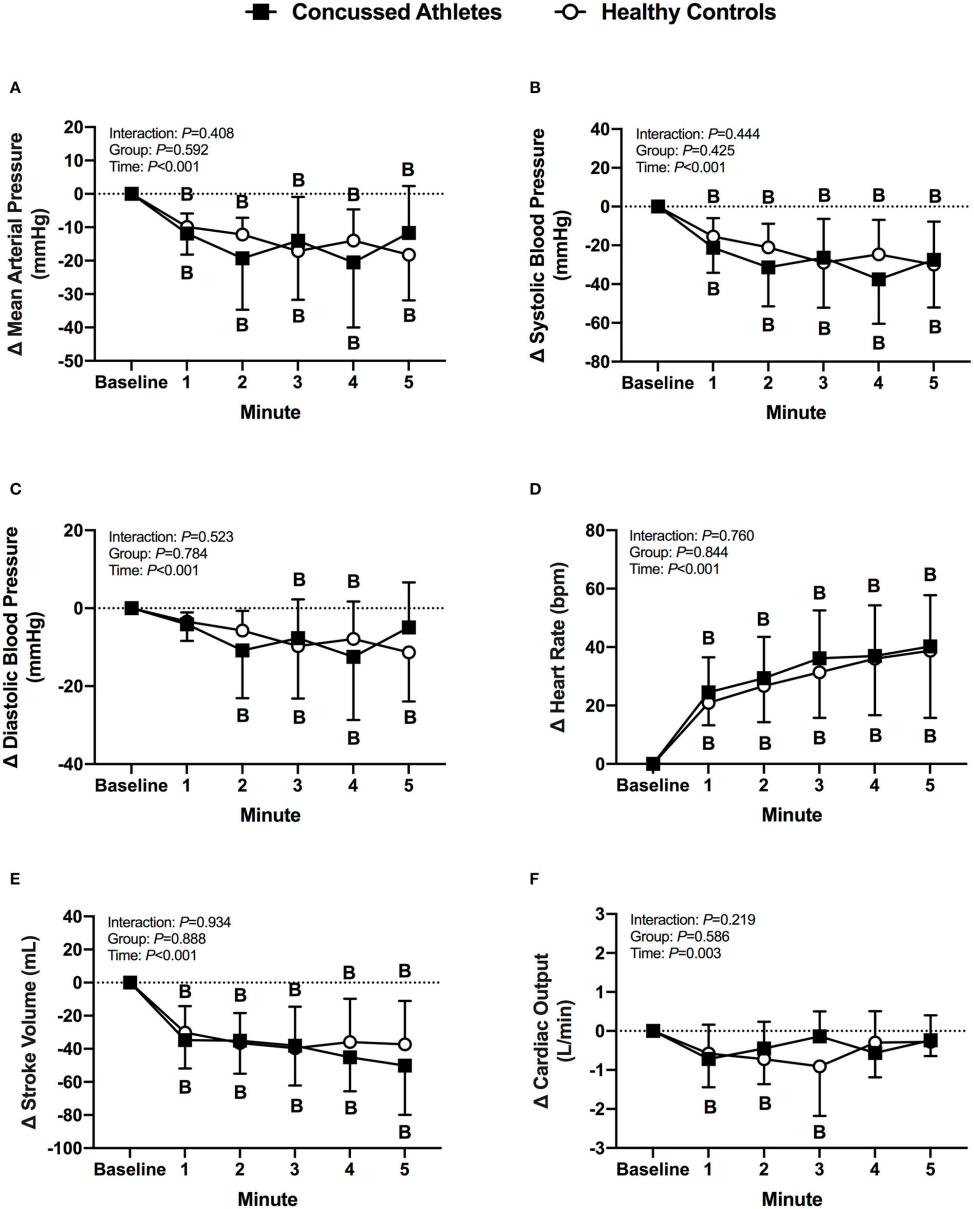
The change from baseline in mean arterial pressure **(A)**, systolic blood pressure **(B)**, diastolic blood pressure **(C)**, heart rate **(D)**, stroke volume **(E)**, and cardiac output **(F)** at each minute of LBNP for concussed athletes (*n* = 10) and healthy controls (*n* = 10). Participants who experienced pre-syncopal signs and did not last the full minute are not included in the statistics for that time point. Concussed athletes were excluded at 3 min (*n* = 4), 4 min (*n* = 5), and 5 min (*n* = 7). Healthy controls were excluded at 3 min (*n* = 1) and 4 min (*n* = 2). B, different from Baseline, (*P* ≤ 0.05).

Mean arterial pressure and systolic blood pressure decreased from baseline in CA (all *P* ≤ 0.006) and HC (all *P* ≤ 0.008) during LBNP. Diastolic blood pressure decreased from baseline in CA at 2 min (*P* = 0.003), 3 min (*P* = 0.055), and 4 min (*P* = 0.003), whereas HC decreased at 3 min (*P* = 0.019), 4 min (*P* = 0.045), and 5 min (*P* = 0.002). Heart rate increased from baseline in CA (all *P* < 0.001) and HC (all *P* < 0.001) during LBNP. Stroke volume decreased from baseline in CA (all *P* < 0.001) and HC (all *P* < 0.001) during LBNP. Cardiac output decreased from baseline in CA at 1 min of LBNP (*P* = 0.014) and decreased in HC at 2 min (*P* = 0.012) and 3 min (*P* = 0.002). The change in cardiovascular variables were not different between groups (*P* ≥ 0.278, Figures 1A–F).

#### Cardiac-Baroreflex Sensitivity

**Figure 2:**
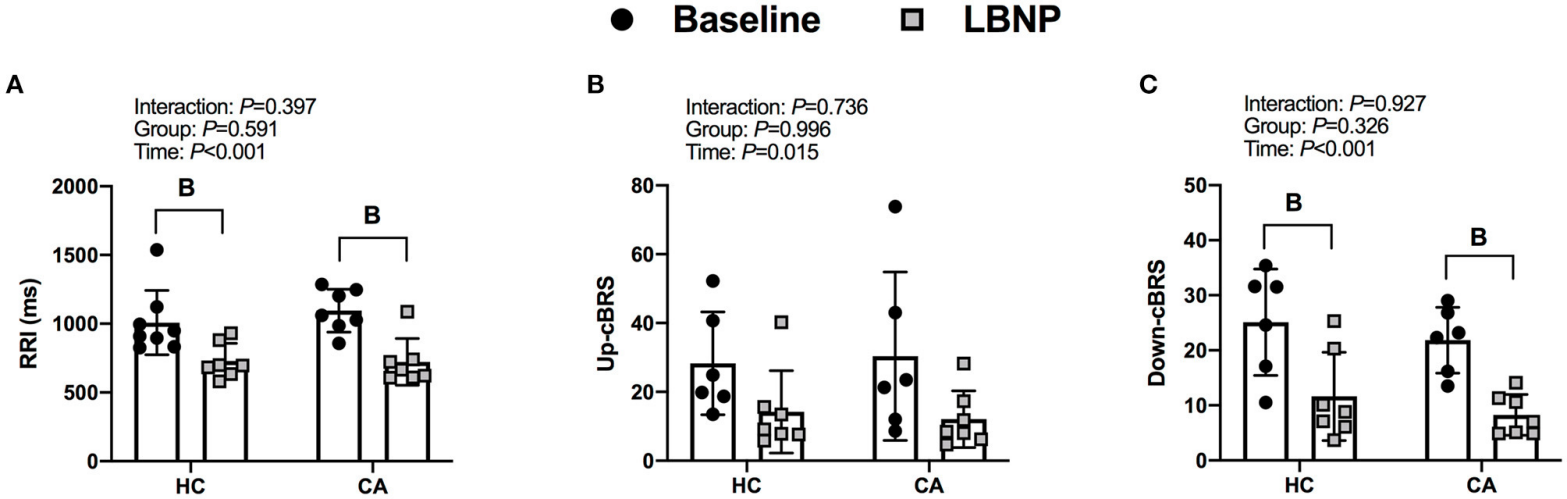
R-R interval **(A)**, Up cardiac- baroreflex senstivity **(B)**, and Down cardiac-baroreflex sensitivity **(C)** for Healthy Controls and Concussed Athletes at baseline and LBNP. B, different from Baseline, (*P* ≤ 0.05).

RRI decreased from baseline in CA (*P* < 0.001) and HC (*P* = 0.002). Up-cBRS had a main effect of time (*P* = 0.015), but *post-hoc*-tests were unable to detect a difference from baseline in CA (*P* = 0.094) or HC (*P* = 0.239). Down-cBRS decreased from baseline in CA (*P* = 0.005) and HC (*P* = 0.005). The change in indices of cBRS were not different between groups (*P* ≥ 0.585, Figures 2A–C).

#### End-Tidal CO_2_ Tension

**Figure 3:**
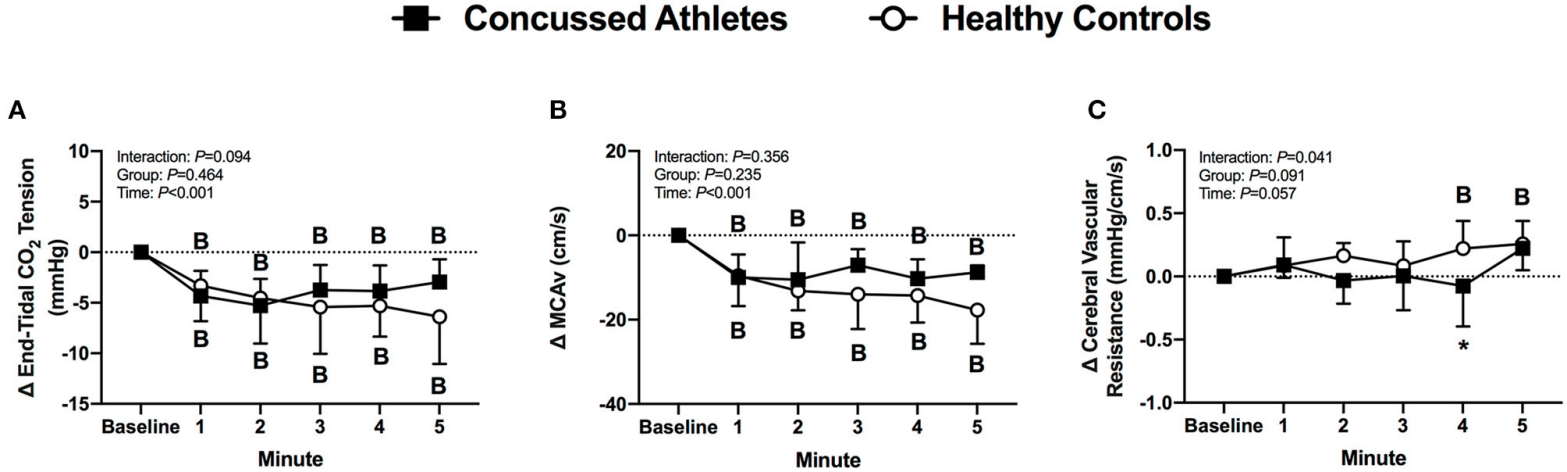
The change from baseline in end-tidal CO_2_ tension **(A)**, MCAv **(B)** and cerebral vascular resistance **(C)** at each minute of LBNP for concussed athletes (*n* = 10) and healthy controls (*n* = 9; one excluded due to poor TCD signal). Participants who experienced pre-syncopal signs and did not last the full minute are not included in the statistics for that time point. Concussed athletes were excluded at 3 min (*n* = 4), 4 min (*n* = 5), and 5 min (*n* = 7). Healthy controls were excluded at 3 min (*n* = 1) and 4 min (*n* = 2). B, different from Baseline (*P* ≤ 0.05). *Different from Healthy Controls (*P* ≤ 0.05).

End-tidal CO_2_ tension decreased from baseline in CA (all *P* ≤ 0.036) and HC (all *P* ≤ 0.003) during LBNP. The change was not different between groups (*P* ≥ 0.246, Figure 3A).

#### Cerebral Vascular Responses

MCAv decreased from baseline in CA (all *P* ≤ 0.003) and HC (all *P* < 0.001) during LBNP, but the change was not different between groups (*P* ≥ 0.431, Figure 3B). Cerebral vascular resistance did not change from baseline in CA (all *P* ≥ 0.730), whereas HC increased at 4 min (*P* = 0.037), and 5 min of LBNP (*P* = 0.008). The change in cerebral vascular resistance was different between groups at 4 min of LBNP (*P* = 0.033, *d* = 1.08, Figure 3C).

#### Total LBNP Time, Survival Rate, and Concussion Symptoms

**Figure 4:**
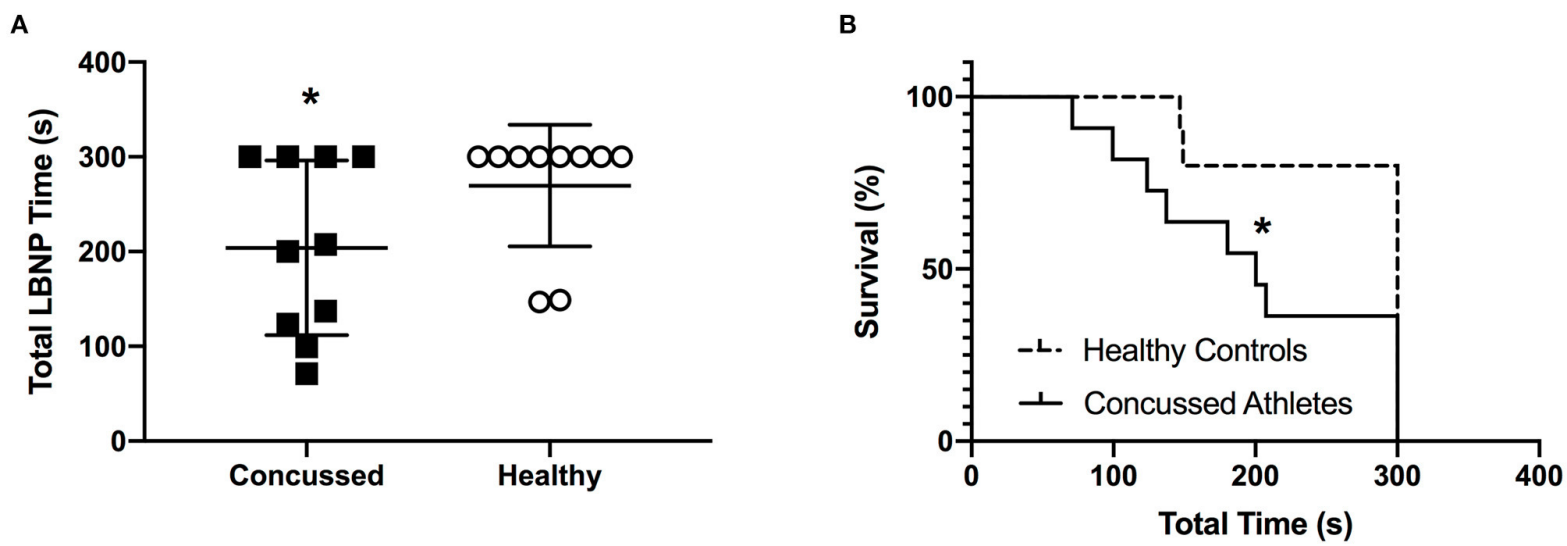
Total LBNP time **(A)** and Kaplan-Meier survival curve **(B)** for Concussed Athletes vs. Healthy Controls. Values are mean ± SD. *Different from Healthy Controls (*P* ≤ 0.05).

**Figure 5:**
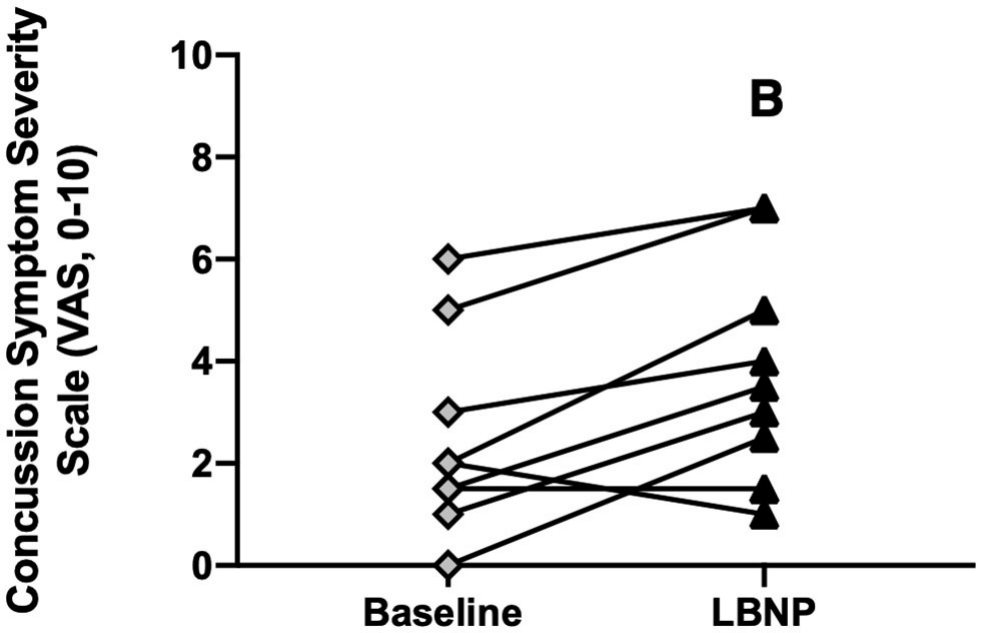
Concussion symptom severity rating (Visual Analog Scale, 0–10) at baseline and after LBNP for concussed athletes (*n* = 10). Values are mean ± SD. B, different from baseline (*P* ≤ 0.05).

Total LBNP time was different between CA (204 ± 92 s) and HC (270 ± 64 s; *P* = 0.040, *d* = 0.83) (Figure 4A). The Kaplan-Meier survival curve with the log-rank (Mantel-Cox) test revealed a median survival time that was different between CA (200 s) and HC (300 s; *P* = 0.049) (Figure 4B). CA experienced an exacerbation of symptoms from pre-LBNP (2 ± 2) to post-LBNP (4 ± 2; *P* = 0.006) (Figure 5).

### Lower Body Positive Pressure

There were no differences between groups for all variables at baseline (Table 1).

#### Cardiovascular Responses

**Figure 6:**
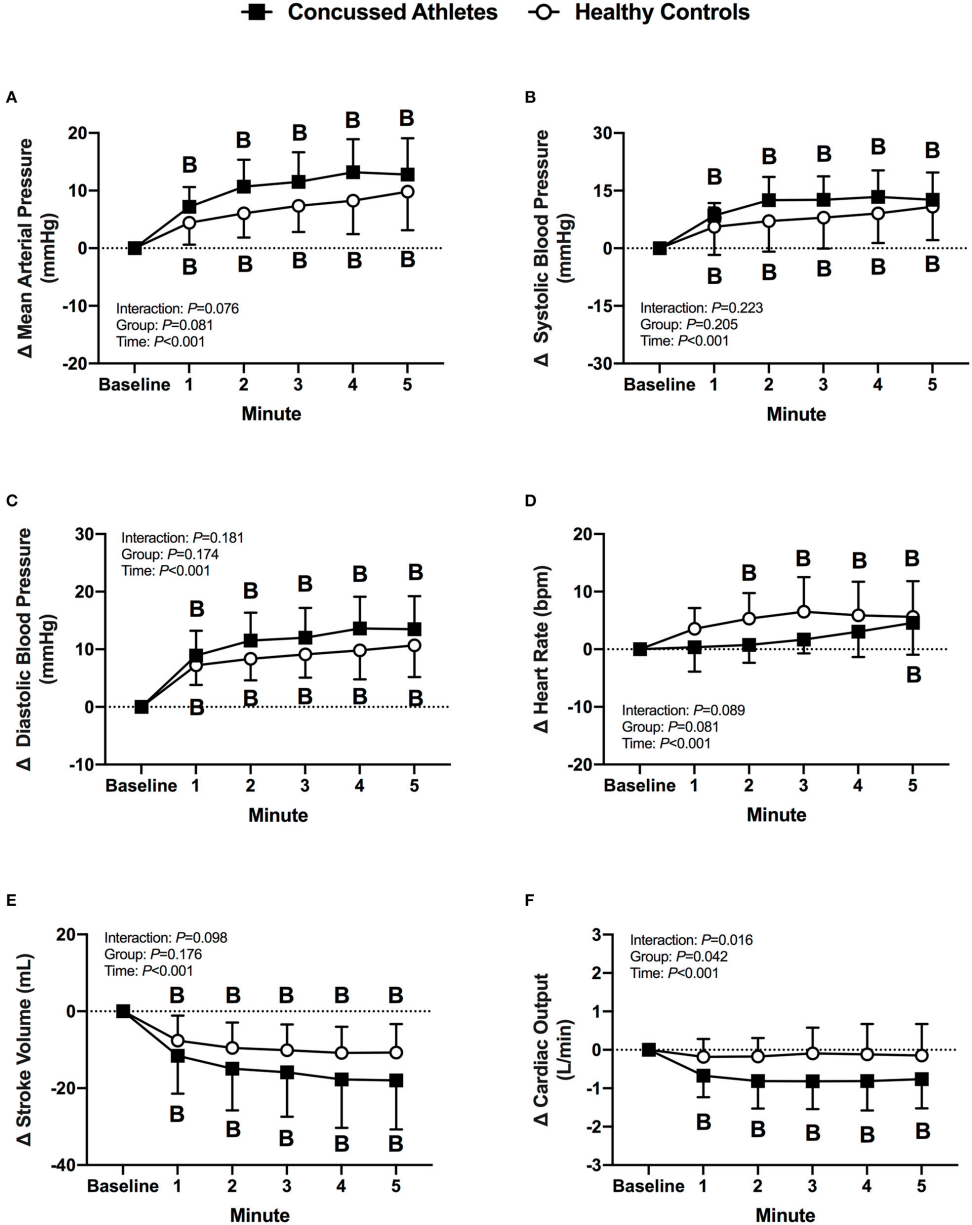
The change from baseline in mean arterial pressure **(A)**, systolic blood pressure **(B)**, diastolic blood pressure **(C)**, heart rate **(D)**, stroke volume **(E)**, and cardiac output **(F)** at each minute of LBPP for concussed athletes (*n* = 10) and healthy controls (*n* = 10). B, different from Baseline (*P* ≤ 0.05).

Mean arterial pressure, systolic blood pressure, and diastolic blood pressure increased from baseline in CA (all *P* < 0.001) and HC (all *P* ≤ 0.008) during LBPP. Heart rate increased from baseline in CA only at 5 min of LBNP (*P* = 0.006), whereas HC had an increase in heart rate at 2 min through 5 min of LBPP (all *P* < 0.001). Stroke volume decreased from baseline in CA (all *P* < 0.001) and HC (all *P* < 0.001) during LBPP. Cardiac output decreased from baseline in CA (all *P* < 0.001) during LBPP, but there was no change from baseline in HC (*P* > 0.999). The change in cardiovascular variables were not different between groups (*P* ≥ 0.064, Figures 6A–F).

#### Cardiac-Baroreflex Sensitivity

**Figure 7:**
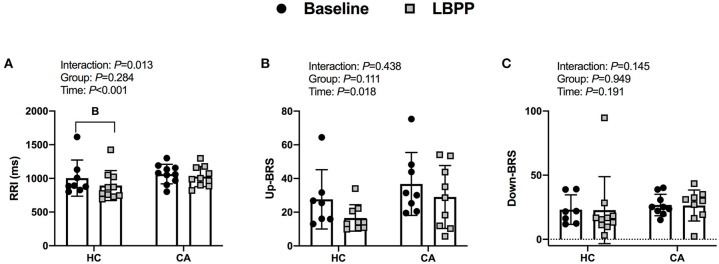
Indices of cardiac-baroreflex sensitivity for Healthy Controls and Concussed Athletes at baseline and LBPP. B, different from Baseline, (*P* ≤ 0.05).

RRI decreased from baseline in HC (*P* < 0.001) but did not change in CA (P = 0.108). Up-cBRS did not change from baseline in CA (*P* = 0.358) or HC (*P* = 0.064). Down-cBRS was not different over time (*P* = 0.191) or between groups (*P* = 0.941). The change in indices of cBRS was not different between groups (*P* ≥ 0.257, Figures 7A–C).

#### End-Tidal CO_2_ Tension

End-tidal CO_2_ tension decreased from baseline in CA at 1 min (*P* = 0.044) and 2 min (*P* = 0.027) of LBPP, whereas HC decreased during LBPP (all *P* ≤ 0.028, Figure 7A). The change in end-tidal CO_2_ tension was not different between groups (*P* ≥ 0.302).

#### Cerebral Vascular Responses

**Figure 8:**
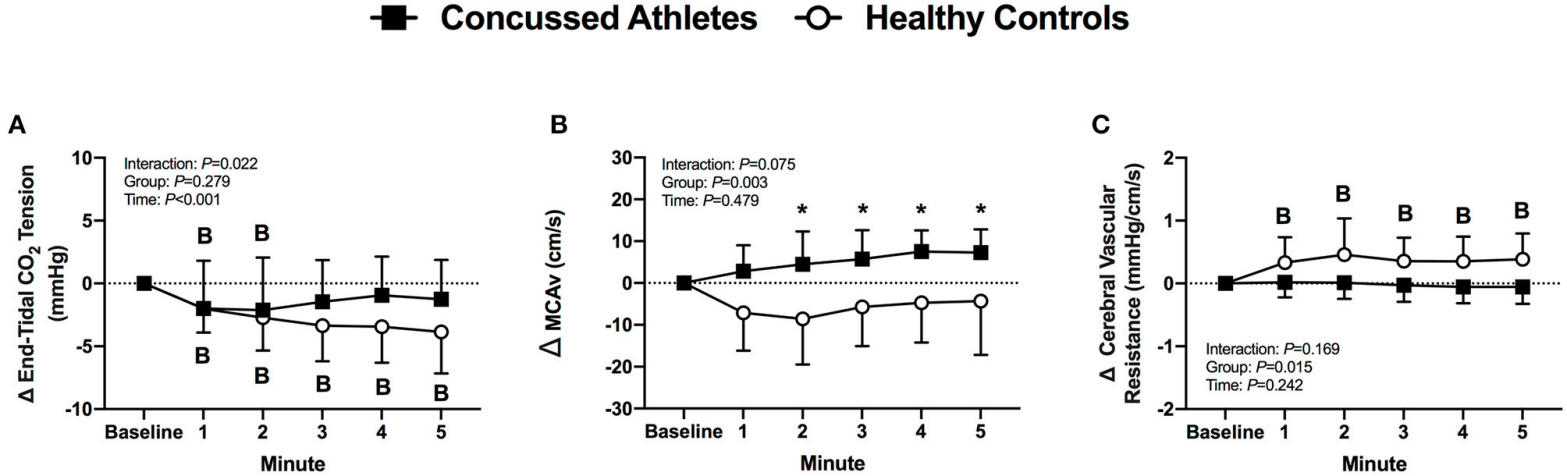
The change from baseline in end-tidal CO_2_ tension **(A)**, MCAv **(B)** and cerebral vascular resistance **(C)** at each minute of LBPP. Values are mean ± SD. B, different from Baseline (*P* ≤ 0.05). *Different from Healthy Controls, (*P* ≤ 0.05).

MCAv did not change in CA (all *P* ≥ 0.193), but decreased in HC at 2 min of LBPP (*P* = 0.039). The change in MCAv was different between groups at 2 through 5 min of LBPP (2 min: *P* = 0.012, *d* = 1.38; 3 min: *P* = 0.039, *d* = 1.39; 4 min: *P* = 0.023, *d* = 1.60; 5 min: *P* = 0.036, *d* = 1.17) (Figure 8B). Cerebral vascular resistance did not change in CA (all *P* > 0.999), but increased in HC during LBPP (all *P* ≤ 0.049). The change in cerebral vascular resistance was not different between groups (*P* ≥ 0.065, Figure 8C).

#### Concussion Symptoms

**Figure 9:**
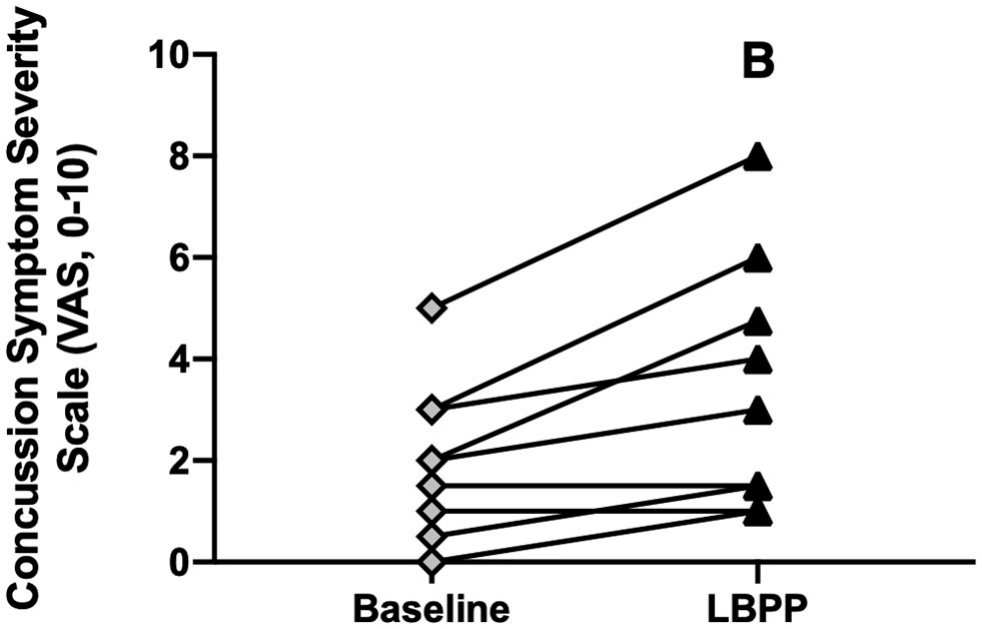
Concussion symptom severity rating (Visual Analog Scale, 0–10) at baseline and after LBPP for Concussed Athletes. Values are mean ± SD. B, different from baseline (*P* ≤ 0.05).

CA experienced an exacerbation of symptoms from pre-LBPP (2 ± 2) to post-LBPP (3 ± 3, *P* = 0.004, Figure 9).

## Discussion

We investigated the cardiovascular and cerebral vascular responses to acute periods of low and high blood pressure in symptomatic concussed college athletes and healthy controls. Our major novel findings are that concussed athletes demonstrated (1) greater orthostatic intolerance during an acute decrease in blood pressure *via* lower total LBNP time due to presyncopal signs and (2) different cerebral vascular responses during an acute increase in blood pressure due to an increase in MCAv compared to healthy controls.

### Lower Body Negative Pressure

During an acute decrease in blood pressure, symptomatic concussed college athletes had significantly lower orthostatic tolerance compared to healthy controls. As expected, a majority of healthy controls tolerated the 5 min LBNP protocol; however, two healthy controls did not complete the protocol due to the onset of presyncopal symptoms. These results are similar to previous findings of relatively low orthostatic tolerance to a moderate LBNP challenge in young, healthy adults (28, 40–42). For example, Xiang et al. found that 33% of young healthy adults were classified as having low LBNP tolerance (42). We found that 50% of the concussed athletes exhibited presyncopal symptoms that reduced total LBNP time compared to healthy controls. The mechanisms that contributed to the low LBNP tolerance in the concussed athletes are not entirely clear based on the cardiovascular and cererbral vascular responses that we observed. We did not find any differences between groups for the blood pressure, cBRS, or MCAv responses (Figures 1–3). However, the healthy controls demonstrated some temporal increases in cerebral vascular resistance whereas the concussed athletes did not (Figure 3C).

The concussed athletes and healthy controls both exhibited reduced cBRS during the LBNP protocol, but we did not observe differences between groups. Previous evidence indicates that a concussion attenuates cBRS. La Fountaine and colleagues found that collegiate males diagnosed with a concussion have reduced spontaneous cBRS at 48 h and 1 wk post-injury compared to healthy matched controls during 5 min of quiet rest (11). Similarly, Hilz et al. reported an attenuation of cBRS in asymptomatic post-mTBI (5–43 months post-injury) during supine rest and upon standing (4). Additionally, it took longer for blood pressure to return to baseline values immediately following a Valsava maneuver in asymptomatic post-mTBI patients (4–98 months post-injury) vs. healthy controls, despite similar cardiovascular responses during the Valsalva maneuver (8). These data indicate altered blood pressure control in both symptomatic and asymptomatic mTBI patients. Our results contradict these previous findings which could be due to methodological differences and/or differences in injury recovery status as we tested within 5 ± 3 days of injury. For instance, La Fountaine and colleagues tested spontaneous cBRS at rest within 48 h and 1 wk of sport-related concussion (11), so it is unclear if baroreflex dysfunction would have been present during a perturbation. To that end, Hilz et al. (8) used postural changes to test asymptomatic post-mTBI patients whereas we tested symptomatic concussed athletes within 10 days of diagnosis using a technique that avoids vestibular activation. Despite our results differing with those of Hilz et al. (8) and La Fountaine et al. (11), animal models that have utilized the lateral fluid percussion brain injury model followed by a phenylephrine pressor test also found no change in baroreflex function within 30 min of the injury (43).

### Lower Body Positive Pressure

The most novel finding from our LBPP protocol was that the healthy controls had an increase in cerebral vascular resistance whereas the concussed athletes did not (Figure 8C). Although statistically insignificant (*P* ≥ 0.065), the change in cerebral vascular resistance throughout LBPP was temporally different in concussed athletes and healthy controls. This was due primarily to lower MCAv responses in the healthy controls despite similar mean arterial pressure responses between groups (Figures 6A, 8B). We speculate that the healthy controls buffered the increase in mean arterial pressure to prevent overperfusion (i.e., increased MCAv) whereas the increased mean arterial pressure in the concussed athletes was likely translated to an increased cerebral perfusion, as indicated by the increase in MCAv during LBPP. Thus, the symptom exacerbation that the concussed atheletes experienced during LBPP could be explained by augmented MCAv. A rise in mean arterial pressure might cause sympathetically-induced cerebral vasoconstriction in order to prevent overperfusion. For instance, Bill and Linder demonstrated that stimulation of the cervical sympathetic chain prevented overperfusion in cats by attenuating cerebral vasodilation (44).

It is possible that other determinants of brain blood flow (e.g., end-tidal CO_2_ tension) could have contributed to the group differences we observed in MCAv during LBPP. Although not statistically different, the concussed athletes had a reduction in end-tidal CO_2_ tension of ~1 mmHg whereas healthy controls exhibited a decrease of ~4 mmHg during LBPP (Figure 8A). Using TCD, it has been shown that cerebral blood velocity decreases by 2% per mmHg decrease in end-tidal CO_2_ tension (45, 46). Hence, it is plasusible that the lower end-tidal CO_2_ tension that we observed might explain why healthy controls had lower MCAv during LBPP vs. the concussed athletes.

We postulate that the increase in MCAv contributed to the exacerbation of symptoms in the concussed college athletes following 5 min of LBPP. Previous evidence indicates a link between symptom severity and elevated regional CBF at rest using arterial spin labeling (6, 47, 48). However, our LBPP protocol augmented mean arterial pressure and MCAv, which provides a better link between cerebral perfusion pressure and concussion symptom severity. In this context, augmented CBF is a normal physiological response to aerobic exercise (49, 50). Patients who have reported persistent post-concussion symptoms exhibited an exacerbated MCAv response to aerobic exercise (51), which is thought to contribute to symptom exacerbation and exercise intolerance following concussion (52–54). However, treadmill exercise testing also activates the vestibular system and could independently increase concussion symptoms (55, 56) whereas the LBPP protocol that we employed provides a more controlled experimental protocol to study the link between increased cerebral blood velocity and concussion symptom exacerbation.

During the LBPP protocol, we observed an increase in heart rate that was temporally different in the concussed athletes and healthy controls. Although not significantly different between groups, healthy controls exhibited an increase in heart rate throughout most of the LBPP protocol (2–5 min) whereas concussed athletes did not (5 min only). We posit that the increase in heart rate along with the rise in mean arterial pressure was due to intramuscular pressure induced by LBPP that activated muscle afferents, which subsequently stimulated the cardiovascular control center. This response has been shown to override the baroreflex mediated decreased heart rate associated with increased blood pressure (29, 57–64). The temporal differences in the heart rate response that could be due to several reasons. First, intramuscular pressure sensitive receptors could be impaired in the concussed athletes. Second, afferent transduction to the cardiovascular control center could be altered in the concussed athletes. Third, the responsiveness of the heart to sympathetic stimulation could be impaired in concussed athletes. We think these reasons are unlikely because we are not aware of peripheral receptors, afferent transduction, and/or end-organ responsiveness to be influenced by a concussion. Therefore, we think there are alterations within the cardiovascular control center within the brainstem of concussed athletes. Although we did not use advanced imaging to determine the location of brain trauma, we have previously found abnormalities in the brainstem of concussed athletes using diffusion tensor imaging (65, 66). Concussion-induced brainstem changes might contribute to altered autonomic control of the cardiovascular system in the concussed athletes. We (67, 68) and others (4, 69–72) have found that autonomic control of the heart during a physioloigcal stressor is altered following concussion and might persist well-beyond symptom resolution (73). Thus, the association among structural brain abnormalities and autonomic function following a concussion warrant further investigation.

### Experimental Considerations

There are several experimental considerations that pertain to the interpretation of our data. First, we did not sport-match the physically active healthy controls to the concussed athletes so there might have been a difference in fitness that contributed to the attenuated LBNP tolerance in the concussed athletes. Highly trained endurance athletes tend to have lower tolerance to orthostatic stress due to structural and mechanical cardiac changes that result in reduced stroke volume due to a steeper slope of the left cardiac pressure-stroke volume relationship (74) or to an altered central venous pressure-central blood volume relation (75) during an orthostatic challenge. However, most of the concussed athletes did not participate in traditional endurance sports (i.e., track, cycling, etc.). We recruited physically active healthy participants who were not currently participating in recreational collision sports to serve as controls in order to avoid the unknown physiological effects of repetitive subconcussive hits on orthostatic tolerance and cerebral vascular function. Second, we only recorded concussion symptom severity before and after each protocol from the concussed athletes. We cannot conclude that the exacerbation of concussion-like symptoms experienced during LBNP and LBPP would not have been experienced by the healthy controls. Thus, it remains unclear if symptom exacerbation following the protocols occurs only in acutely concussed athletes or if it is a normal response to experiencing a brief period of central hypo- and hypervolemia. The visual analog symptom severity scale also does not provide information on categorical symtpoms (e.g., headache, dizziness, nausea). Therefore, we cannot provide insight whether only pre-testing symptoms were exacerbated throughout the test, if new symptoms arose from pre- to post-testing, or if categorical symtpoms differed between the protocols. Third, TCD is a non-invasive tool used to measure beat-to-beat cerebral blood velocity of various intra-cranial arteries, primarly to assess responses to changes in arterial CO_2_ and/or blood pressure. TCD does not provide a measure of artery diameter and thus the assumption is made that changes in cerebral blood velocity directly reflect changes in CBF (i.e., artery diameter remains constant). Giller et al. reported small changes in MCA diameter (~2-2.5%) with alterations in arterial CO_2_ (±10 mmHg) and blood pressure (±18 mmHg) during craniotomy (76). On the other hand, MRI data revealed constant MCA diameter during a hypercapnia protocol that increased PETCO_2_ by ~8 mmHg (77). We cannot confirm if the changes observed in MCAv are due to alterations in artery diameter or cause changes in cerebral blood flow. Fourth, we did not clamp end-tidal CO_2_ tension to match baseline values. Thus, we cannot draw conclusions on the mechanisms that contributed to the MCAv during decreases and increases in blood pressure since end-tidal CO_2_ tension was not constant throughout the protocols. Although this approach reduces our capability to fully understand changes in cerebral blood flow regulation, we think it does enhance our ability to understand the physiology of concussion patients outside of the laboratory (e.g., greater external validity). Fifth, women were tested at different phases of the menstrual cycle due to our criteria of testing within the first 10 days of injury while symptomatic. It is possible that menstrual cycle phase influenced our findings. Sixth, because of the relatively large number of early LBNP terminations in the CA group, we have a low number of participants for some of the statistical comparisions. Lastly, we did not reassess CA after they were clinically cleared to return to play. This approach would have allowed us to determine if CA returned to physiological function that is exhibited by healthy controls prior to their return to play. Important emerging evidence indicates that physiological dysfunction persists in concussed patients beyond symptom resolution (4, 8, 73).

### Perspectives and Significance

Recently concussed athletes can exhibit signs of exercise intolerance (78). There is mounting evidence that baroreflex dysfunction and altered cerebral vascular function are present following a concussion, which might contribute to exercise intolerance. At rest and during exercise, systemic blood pressure and intracranial pressure control are critical for maintaining blood flow to meet cerebral oxygen demand. The brain is susceptible to hypo- and/or hyperperfusion if systemic blood pressure (i.e., baroreflex) and/or intracranial pressure (i.e., cerebral autoregulation) control is/are impaired. In the present study, we assessed the cardiovascular and cerebral vascular responses to acute increases and decreases in blood pressure in symptomatic concussed college athletes vs. recreationally active healthy controls. Our data suggest a greater prevalence of orthostatic intolerance and different MCAv responses during the first 10 days after sustaining a sport-related concussion, both of which were associated with symptom exacerbation. These findings provide insight into the mechanism(s) for concussion symptoms (e.g., dizziness and headache) as these protocols simulate the blood pressure responses to postural changes (i.e., sit-to-stand) and aerobic exercise without activating the vestibular system. Although we recognize that more research is warranted, the prevalence of orthostatic intolerance observed in symptomatic concussed athletes demonstrates that examining the physiological response to a stressor might be an additional and useful tool to assist clinicians with their return-to-play decisions.

### Conclusion

Although our LBNP protocol was not designed to test orthostatic tolerance, we found that symptomatic concussed athletes experienced attenuated orthostatic tolerance compared to recreationally active healthy controls. Additionally, LBPP induced similar changes in mean arterial pressure and end-tidal CO_2_ tension between groups, however MCAv was elevated only in symptomatic concussed athletes which could have contributed to concussion symptom exacerbation.

## Data Availability Statement

The raw data supporting the conclusions of this article will be made available by the authors, without undue reservation.

## Ethics Statement

The studies involving human participants were reviewed and approved by University at Buffalo Institutional Review Board. The patients/participants provided their written informed consent to participate in this study.

## Author Contributions

ZS, BW, JL, and BJ conceived the study and designed the experiment. MO'L, JS, ZS, and BJ performed experiments and collected the data. MW, MO'L, and JS extracted and analyzed data. MW and BJ performed statistical analyses. MW drafted the manuscript. MW, MO'L, JS, ZS, BW, JL, and BJ edited and approved the final manuscript. All authors contributed to the article and approved the submitted version.

## Conflict of Interest

The authors declare that the research was conducted in the absence of any commercial or financial relationships that could be construed as a potential conflict of interest.
